# Variables associated with speech and language therapy time for aphasia, apraxia of speech and dysarthria

**DOI:** 10.1590/1980-57642018dn13-010007

**Published:** 2019

**Authors:** Maysa Luchesi Cera, Tatiana Piovesana Pereira Romeiro, Patricia Pupin Mandrá, Marisa Tomoe Hebihara Fukuda

**Affiliations:** 1 Speech and Language Therapist, PhD, Professor, Faculdade de Ceilândia, Universidade de Brasília. Brasília, DF, Brazil.; 2 Speech and Language Therapist, Faculdade de Medicina de Ribeirão Preto, Universidade de São Paulo. Ribeirão Preto, SP, Brazil.; 3 Speech and Language Therapist, PhD, Professor, Faculdade de Medicina de Ribeirão Preto, Universidade de São Paulo. Ribeirão Preto, SP, Brazil.

**Keywords:** rehabilitation, communication, speech, language and hearing sciences, aphasia, dysarthria, reabilitação, comunicação, fonoaudiologia, afasia, disartria

## Abstract

The most common acquired communication disorders are aphasia, dysarthria and apraxia of speech. Determining the factors associated with speech therapy time of adults with these disorders can further the understanding of the speech and language rehabilitation process within the public service and linked to the health education process.

**Objective:**

To analyze the types of acquired neurological disorders of communication of patients treated during the first years of implementation of a medium complexity service, along with demographic data, and rehabilitation time; and to determine associations between rehabilitation time and age, education, type of communication disorder, neurological disease duration and having been seen by a trainee.

**Methods:**

A retrospective analysis of the records of patients with acquired neurological disorders of communication who started speech and language rehabilitation between 2010 and 2011 was performed.

**Results:**

A total of 86 cases with acquired disorders of communication were seen, of whom 66% had aphasia, 35% dysarthria and 26% apraxia of speech. Mean age was 59 years and stroke was the most frequent cause (71%). Fifty patients completed speech-language rehabilitation and had a mean therapy time of 12 months. Aphasia and apraxia of speech were associated with a longer rehabilitation time. Therapy time until discharge was not significantly associated with lesion duration, education, age or being seen by a trainee.

**Conclusion:**

The duration of speech therapy for acquired neurological disorders of communication is long and associated with the type of disorder.

## RESUMO.

Os transtornos adquiridos da comunicação mais prevalentes são as afasias, as disartrias e as apraxias de fala. Compreender os fatores associados ao tempo de atendimento em terapia da fala de adultos com estes transtornos contribuirá para o conhecimento do processo de reabilitação fonoaudiológica inserido no serviço público vinculado ao processo de ensino em saúde.

**Objetivo:**

Analisar os tipos de transtornos neurológicos adquiridos da comunicação, os dados demográficos e o tempo de terapia fonoaudiológica de usuários atendidos nos dois primeiros anos de implantação de um serviço público brasileiro de reabilitação. Verificar a associação entre o tempo de reabilitação e a idade, a escolaridade, o tipo do transtorno da comunicação, o tempo de doença neurológica e ter sido atendido por estagiário.

**Métodos:**

Análise retrospectiva de prontuários dos usuários que passaram pelo primeiro atendimento antes da implantação de regras institucionais que limitavam o tempo máximo de tratamento.

**Resultados:**

Foram atendidos 86 usuários, dos quais 66% possuíam afasia, 35% disartria e 26% apraxia de fala. A idade média foi de 59 anos e o acidente vascular cerebral foi a causa mais frequente (71%). Dos cinquenta que seguiram a reabilitação até a alta fonoaudiológica, o tempo médio de terapia foi de 12 meses. Ter afasia ou apraxia de fala foram variáveis associadas a um maior tempo de reabilitação. O tempo de lesão, os fatores sociodemográficos e ser atendido por estagiário não estiveram associados ao tempo de terapia.

**Conclusão:**

O tempo médio de terapia fonoaudiológica para os transtornos adquiridos da comunicação é longo e está associado ao tipo de transtorno apresentado.

The most common acquired disorders of communication following injury to areas of the nervous system are aphasia, dysarthria and apraxia of speech. Aphasia is as an acquired impairment of language processes underlying receptive and expressive modalities that is caused by damage to certain areas of the brain which are primarily responsible for the language function.^1^ Dysarthria is a speech disorder resulting from a weakness, paralysis, or incoordination of the speech musculature that is of neurological etiology.^2^ Apraxia of speech is a phonetic-motor disorder of speech production, which results in intra- and inter-articulator temporal and spatial segmental and prosodic distortions, not attributable to primary deficits in sensory or language information processing.^3^

In Brazil, the most common causes of brain lesions associated with these communication disorders are stroke and traumatic brain injury (TBI).^4^ The annual incidence of stroke in a Brazilian city was 108 cases per 100,000 population.^5^ A Canadian study reported an incidence, in patients after ischemic stroke, of 30% aphasia and 42% of dysarthria.^6^ In a Brazilian rehabilitation service for acquired communication disorders, aphasia was the most prevalent (56%), followed by dysarthria (33%) and apraxia of speech (19%).^4^ In this same service, the duration of speech and language therapy was typically up to 6 months.^4^

The Integrated Center for Rehabilitation of the State Hospital of Ribeirão Preto (CIR-HERP) is a public rehabilitation unit set-up in 2009. Since 2010, the center has delivered around 30 weekly sessions for acquired communication disorders. Akin to many Brazilian speech and language rehabilitation services, therapy sessions for patients are provided weekly and last 45 minutes. Ribeirão Preto, the city where the CIR-HERP is located, is home to the Regional Department of Health, which handles cases from catchment areas covering 26 cities and a population of 1,327,989 inhabitants.^7^ Given that it is affiliated with the Faculdade de Medicina de Ribeirão Preto (FMRP), the sessions are also conducted by trainees or speech language preceptors.

Up until the beginning of 2012, the treatment time of patients of the service depended on the subjective and individual analysis of the assisting therapist, without restriction to the number of sessions or minimum score for cognitive or functional improvement. The growing demand for the speech and language rehabilitation service prompted this study, performed to aid the planning of treatment outcome indicators. The results of this study can help establish speech-language therapy time and planning of criteria for regular reassessment to define treatment protocols. The findings of this study can inform health managers of other services providing treatment for cases with acquired communication disorders.

Knowing the treatment time of adults and elderly with acquired communication disorders and the factors associated with this parameter is key to understanding the speech and language rehabilitation process within the public health service and linked to the health education process.

In Brazil, the knowledge about rehabilitation for acquired neurological communication disorders held by the population remains lacking, a factor which often explains the patient delays in seeking rehabilitation services. Given that Brazil is a developing country with major sociodemographic diversity, it is important to determine whether age and education are associated with time between start of speech and language therapy and discharge from the treatment. Age^8^^-^ 9 and education effects on cognitive performance have been frequently reported.^10^ A Brazilian study showed that healthy illiterate subjects had worst language and cognitive performance relative to literate subjects.^11^ Parente et al. (2008) discussed that multiple interactions with the environment lead the brain to function in an adaptive form. The authors reported literacy as a determining factor for brain organization and suggested that cerebral language representation seems to be more bilateral in illiterate individuals than in educated individuals.^12^ However, no studies were found on the association between these sociodemographic variables and therapy time of subjects with acquired disorders of communication. Thus, in view of the evidence of worst cognitive performance among older and lower-educated subjects, these variables are believed to be associated with longer speech and language therapy time.

The presence of cognitive impairment was associated with a longer follow-up, of up to 4 years after stroke.^13^ Therefore, in the present study, a longer follow-up time was expected, according to the type of communication disorder, especially for cases of aphasia or apraxia with cognitive deficits. Also, rehabilitation time was expected to be associated with lesion duration and with treatment provided by trainees.

Therefore, the objectives of this study were: 1) to describe acquired neurological disorders of communication, speech-language rehabilitation time and demographic data of patients of a public healthcare service; 2) to determine the association between speech-language treatment time and age, education, type of communication disorder, lesion duration and having been seen by a trainee.

## METHODS

A cross-sectional observational study was conducted based on a retrospective analysis of medical records of patients of the Sector for acquired neurological disorders treated by the speech therapy service of the CIR-HERP between January 2010 and December 2011. The management boards of the CIR-HERP and FMRP authorized the study. This research project was approved by the Ethics Committee of the Faculdade de Medicina de Ribeirão Preto under permit number 2.017.401 and CAAE 66805317.9.0000.5440.

Inclusion criteria were: age >18 years, patient having attended the CIR-HERP for speech and language therapy, and presenting with acquired neurological disorder of communication due to stroke, TBI, cognitive-behavioral neurodegenerative disease, neuromuscular disease or other acquired neurological disease. The exclusion criteria were: having a developmental speech or language disturbance, and not having an acquired neurological disorder of communication.

The sample comprised all cases of the CIR-HERP who started speech therapy at the Sector for acquired neurological disorders between 2010 and 2011, the implementation period of the service. The rehabilitation of the cases was based predominantly on the principles of the cognitive neuropsychology school and on the use of a range of different approaches and therapeutic methods for disorders of communication. All patients received homework involving daily practice of therapeutic activities, but no monitoring of its application was carried out. For cases with neurodegenerative disease, the therapeutic objectives were proposed for immediate adaptations and, after discharge from medium-complexity care, all patients remained in follow-up at the basic healthcare service.

All information was collected from the medical records of these patients, as well as for cases registered after 2011, in cases discharged from speech language therapy after this period. After the initial appointment of the patient, return visits were weekly and sessions lasted around 45 minutes each.

Data characterizing the sample were gathered, such as: age at date of first assessment; sex; education (full years of study); profession; lesion duration in months (time elapsed between date of lesion and first speech-therapy assessment at the service); neurological cause (stroke, TBI, cognitive-behavioral neurodegenerative disease, neuromuscular disease or other neurological disease); presence of aphasia, apraxia of speech or dysarthria; presence of dysphagia associated with communication disorder; speech therapy time (in months, from admission to service to date of discharge); final conduct (discharge having met objectives, discharge for having reached therapy limit, discharge due to drop-out or referral); and whether seen by a graduate or student specializing in speech-therapy. Discharge due to drop-out was defined when the patient failed to attend three consecutive sessions without explanation or when they informed the service that they would not be resuming treatment, for personal reasons, directly or indirectly related to the rehabilitation. When the therapy was delivered by a student, this therapist was replaced by another trainee after one year.

Version 22 of IBM SPSS *Statistics* was employed for descriptive and inferential statistical analysis. The distribution of variables was tested using the Shapiro-Wilk test. The dependent variable exhibited a non-normal distribution and therefore Spearman’s correlation test was used to determine the association between therapy time and age, education and lesion duration. The contingency test was also performed to verify the association between therapy time and the variables: sex, type of lesion, type of communication disorder (aphasia, dysarthria and apraxia of speech) and having been seen by a student.

## RESULTS

### General characteristics of sample

During the study period from January 2010 to December 2011, 89 cases were seen at the Adult Speech-Language sector of the CIR-HERP. Three patients were excluded from the study for having developmental language disorders.

Of the 86 patients with acquired neurological disorder of communication, 58% were male. Regarding professions, 38% were retired prior to sustaining the lesion and, of the 62% that worked or were on sick leave, 21% were homemakers or housemaids, 12% salespersons, 6% factory workers, 5% drivers, 4% farm laborers or engaged in other professions.

Mean age of the 86 cases seen was 59±15 (25-91) years, education 6±4 (0-17) years, lesion duration 48±84 (1-420) months, while mean treatment time at the Speech-Language sector was 8±7 (1-37) months.

The most common cause of neurological impairment was stroke, accounting for 71% of cases. A further 12% had a neuromuscular disease (Parkinson´s disease, spinocerebellar ataxia, among others), 7% were diagnosed with dementia, 6% had TBI, 3% neurotoxoplasmosis and only 1% had meningioma.

The three communication disorders presented by the patients of the sector between 2010 and 2011 were aphasia (66%), dysarthria (35%) and apraxia of speech (26%). Thirteen (15%) individuals also had dysphagia which co-occurred with the disorders of communication. All cases with apraxia of speech co-occurred with aphasia and only 20% of the patients with dysarthria also had aphasia. Apraxia of speech and dysarthria did not co-occur in this study.

### Speech and language therapy time

Of the 86 subjects in the sample, 50 (58%) underwent the rehabilitation process until discharge from speech-language therapy, 27 (31%) dropped out of the follow-up during the assessment or rehabilitation process and nine (11%) were referred to a tertiary care service.

A total of 50 patients completed the rehabilitation process at the service until discharge and had a mean therapy time of 12±8 (1-37) months.

### Association between therapy time and sociodemographic, neurologic and service-related variables

The variables associated with speech and language therapy time for acquired neurological disorders of communication are shown in Tables 1 and 2. Therapy time was associated with the type of disorders of communication.

**Table 1:** Association between therapy time and age, education and lesion duration

	Spearman's correlation coefficient	*p
Age	-0.31	0.831
Education	0.01	0.955
Lesion duration	-0.16	0.262

* p: statistically significant.

**Table 2:** Association between therapy time and profession, sex, lesion type, presence of aphasia, apraxia of speech, dysarthria or dysphagia and having been seen by trainee.

		N*	Mean	SD^+^	Minimum and maximum	χ^2§^	p^||^	C^¶^
Sex	M**	30	11.5	7.88	3-37	28.07	0.108	0.60
	F^++^	20	11.85	8.11	1-34			
Lesion type	Stroke	34	12.47	8.23	1-37	81.6	0.910	0.79
	Others	16	9.88	7.04	1-24			
Aphasia	Yes	14	12.86	7.40	04-30	31.53	**0.049**	0.62
	No	36	11.17	8.13	01-37			
Aphasia with apraxia of speech	Yes	14	16.86	8.32	07-37	36.52	**0.013**	0.65
	No	36	9.61	6.81	01-30			
Aphasia with dysarthria	Yes	04	6.75	2.06	04-09	15.71	0.734	0.49
	No	46	12.06	8.08	01-37			
Dysarthria	Yes	18	7.72	6.20	01-23	32.01	**0.043**	0.63
	No	32	13.84	7.96	04-37			
Dysphagia	Yes	7	8.86	5.61	01-14	15.16	0.767	0.48
	No	43	12.09	8.16	01-37			
Seen by trainee	Yes	14	16.14	9.03	05-37	30.66	0.060	0.62
	No	36	9.89	6.75	01-30			

* N: number;

+ SD: standard deviation;

§ χ^2:^ Chi-square test;

|| p: statistically significant;

¶ C: contingency test;

** M: male;

++ F: female.

## DISCUSSION

The present study found that mean speech-language therapy time for acquired neurological disorders of communication was long, averaging 12 months, and was associated with the presence of aphasia or apraxia of speech. However, therapy time until discharge was not significantly associated with lesion duration, education, age or being seen by a trainee.

In this study, speech-language therapy time for acquired neurological disorders of communication was longer than that reported in the study by Talarico et al. (2011), in which most patients were treated for 6 months.^4^ This longer therapy time might be related to the greater prevalence of aphasia and apraxia of speech in the present study population compared with the cited study, disorders which are associated with longer rehabilitation time (Table 2). Previous studies have shown an association between presence of cognitive impairment and longer post-stroke follow-up.^13^ Therefore, the present study revealed that the cognitive deficits of aphasia and apraxia of speech were associated with the need for longer treatment time to improve communication. In addition, having dysarthria was statistically associated with shorter therapy time.

The disparities in mean speech language therapy time among different Brazilian public health services may also be related to neurological variables, such as lesion duration. In the present study, mean time to neurological diagnosis and start of treatment, defined as lesion duration, was 48 months. The study conducted in the tertiary care setting did not report lesion duration of patients at start of treatment, but the authors discussed the importance, for rehabilitation, of commencing therapy within the first few months after lesion.^4^ In general, patients of the tertiary care services, whose team includes a speech therapist, begin the therapy process from the time of hospital admission, allowing rehabilitation to be started earlier. Patients of the medium-complexity services often begin the rehabilitation process only after discharge from tertiary care, are not always given early speech therapy guidance and typically spend long periods on waiting lists for therapy. The present results showed that lesion duration varied greatly among patients, ranging from 1 to 420 months, but no association with speech therapy time was evident on the correlation test (Table 1). The absence of an association is likely explained by the fact that only 11 (22%) cases started the rehabilitation process within six months of the lesion. Thus, the results concerning association between lesion time and therapy time should be considered for patients with delayed rehabilitation. Although these results were not explored in the present study, there is believed to be an association between lesion time and therapy time for cases with early intervention.

Another neurological variable assessed was cause of the communication disorder. The most frequent cause was stroke, accounting for 71% of service patients, corroborating the finding of the study by Talarico et al. (2011), in which 69% of cases of a tertiary speech-language therapy service had this cerebrovascular disease.^4^ However, only 7% of the study sample had dementia. Despite the disease´s growing incidence, this result indicates that few cases of neurodegenerative disease whose initial symptoms are cognitive have access to therapy, in contrast to that observed for patients post-stroke. The increase in referral of patients with dementia to communication therapy services may change the present study results with regard to the absence of an association between treatment time and type of neurological disease.

Thus, the disparities in length of treatment of patients of different Brazilian public health services might be related to a number of factors, beyond the complexity (medium or high) of the health care. It is important to recognize that each service has other specific variables which may be associated with speech-language therapy time. The present study reported the main general variables, present in any rehabilitation service, namely, age, education, sex, lesion duration and type of communication disorder. A differential of the present study was the failure to find an association between treatment time for communication disorders and being seen by a trainee, a characteristic inherent to services run in partnership with universities.

Being seen by an undergraduate student supervised by a preceptor and a professor was not associated with longer rehabilitation time (Table 2). It is fundamental to emphasize that the presence of trainees allows an overall greater number of sessions to be given because, while a preceptor can see only one case at a time, more patients are seen within the same timeframe when a group of trainees under supervision is deployed. In addition, the presence of students undergoing training requires constant updating of the team, with consequent improvements in the quality of the service.

Regarding the variables associated with rehabilitation time, the results of this study failed to show an association with age or education. Cao et al. (2007) observed a positive correlation between educational level and cognitive performance of young adults aged 18-47 years that suffered strokes,^14^ and hence there was expected to be an association between education and therapy time, a relationship not found in the present study. No previous studies associating treatment time for neurological disorders with sociodemographic factors were available. It is important to note that the absence of an association between and education and rehabilitation time should be interpreted mainly for patients with low educational level, a characteristic of this sample.

Regarding age, half of the sample comprised adults aged ≤59 years and half elderly. Therefore, the absence of an association between therapy time and age corroborated the presence of neuroplasticity, independent of age.

Notably, some variables were not assessed in this study, but should be considered in clinical practice, such as the presence of changes in behavior or another cognitive function, the therapeutic approach for communication therapy, patient adherence in homework completion, communication disorder severity, the stages of language and speech processing affected, and the presence of family support. The results of this study can support services that treat acquired disorders of communication and help inform speech therapists on the expected time required for the rehabilitation process, thereby guiding the patient and planning of the service.

Individuals with acquired neurological disorders of communication require rehabilitation involving a long period of speech language therapy averaging 12 months. Having aphasia or apraxia of speech was statistically associated with longer rehabilitation time. Having dysarthria was statistically associated with shorter therapy time, whereas other variables, such as lesion duration, education, age and being seen by a trainee showed no such association.
